# Association between rs2107595 HDAC9 gene polymorphism and advanced carotid atherosclerosis in the Slovenian cohort

**DOI:** 10.1186/s12944-020-01255-1

**Published:** 2020-04-13

**Authors:** Emin Grbić, Nataša Gorkič, Aleš Pleskovič, Marjeta Zorc, Farid Ljuca, Mladen Gasparini, Božidar Mrđa, Ines Cilenšek, Sara Mankoč, Maciej Banach, Daniel Petrovič, Zlatko Fras

**Affiliations:** 1 Department of Physiology, Faculty of Medicine, University of Tuzla, Tuzla, Bosnia and Herzegovina; 2 International Center for Cardiovascular Diseases MC Medicor d.d, Izola, Slovenia; 3 Department of Cardiology, Division of Medicine, University Medical Centre of Ljubljana, Ljubljana, Slovenia; 4 Department of Vascular Surgery, General Hospital Izola, Izola, Slovenia; 5 Department of Vascular Surgery, University Medical Centre Maribor, Maribor, Slovenia; 6 Institute of Histology and Embryology, Faculty of Medicine, University of Ljubljana, Vrazov trg 2, SI-1000 Ljubljana, Slovenia; 7 Cardiovascular Research Centre, University of Zielona-Gora, Zielona Gora, Poland; 8 Polish Mother’s Memorial Hospital Research Institute (PMMHRI), Lodz, Poland; 9 Division of Medicine, Centre for Preventive Cardiology, Division of Medicine, University Medical Centre Ljubljana, Zaloška cesta 7, SI-1525 Ljubljana, Slovenia; 10 Chair of Internal Medicine, Medical Faculty, University of Ljubljana, Ljubljana, Slovenia

**Keywords:** Histone deacetylase 9 (HDAC9) gene polymorphism, Atherosclerosis, Carotid atherosclerosis, Advanced carotid atherosclerosis

## Abstract

**Background:**

Histone deacetylase 9 (HDAC9) plays an important role in transcriptional regulation, cell cycle progression and developmental events; moreover, it has been investigated as a candidate gene in a number of conditions, including the onset and progression of atherosclerosis. We hypothesized that the rs2107595 *HDAC9* gene polymorphism may be associated with advanced carotid artery disease in a Slovenian cohort. We also investigated the effect of this polymorphism on HDAC9 receptor expression in the internal carotid artery (ICA) specimens obtained by endarterectomy.

**Methods:**

This case-control study enrolled 619 unrelated Slovenian patients: 311 patients with ICA stenosis > 75% as the study group and 308 patients with ICA stenosis < 50% as the control group. Patient laboratory and clinical data were obtained from the medical records. The rs2107595 polymorphisms were genotyped using TaqMan SNP Genotyping assay. HDAC9 expression was assessed by immunohistochemistry in 30 ICA specimens from patients with ICA atherosclerosis > 75%, and the numerical areal density of HDAC9 positive cells was calculated.

**Results:**

The occurrence of advanced ICA atherosclerosis in the Slovenian cohort was 3.81 times higher in the codominant genetic model (OR = 3.81, 95%CI = 1.06–13.77, *p* = 0.04), and 3.10 times higher in the recessive genetic model (OR = 3.10, 95%CI = 1.16–8.27, *p* = 0.02). In addition, the A allele of rs2107595 was associated with increased HDAC9 expression in the ICA specimens obtained by endarterectomy.

**Conclusions:**

We observed a significant association between the AA genotype of rs2107595 with the advanced carotid artery disease in our Slovenian cohort, indicating that this polymorphism may be a genetic risk factor for ICA atherosclerosis.

## Introduction

Atherosclerosis is a chronic inflammatory disease characterized by the accumulation of fat, cholesterol, calcium and other substances from the blood into the inner layers of arterial blood vessels of various size. Atherosclerosis related vascular disease (ASCVD) can lead to different clinical manifestations. These are related to the vascular bed mostly affected, and among the most dangerous are the atherosclerotic complications within the coronary or carotid arteries, manifested as either the heart attack or stroke, respectively [1].

Carotid atherosclerosis is among the major causes of stroke and transient ischemic attacks (TIA). An increase in the stenosis of the internal carotid artery diameter of more than 50% is associated with about 15% of cases of ischemic stroke (IS) [2].

Stroke rates differ around the world; for example, from 9 per 100,000 in Qatar and 138 per 100,000 people in Russia. The average stroke death rate in Europe is 96 per 100,000 people, while in North America it is 19 per 100,000 people per year [3]. It has also been observed that the mortality rate increases with age and varies in highly and poorly developed economies [4]. As the number of patients with ASCVD increases with age, so do the effects it has on the quality of life of people with the disease. Therefore, more research is needed to prevent and slow the progression of atherosclerosis.

It is generally accepted that epigenetic and genetic factors play an important role in the onset and progression of ASCVD [5–10]. An important role in epigenetic activity plays the HDAC9 enzyme, encoded by the Histone Deacetylase 9 (*HDAC9)* gene, which affects the chromosomal structure and performs histone deacetylation by inhibiting transcription [11]. *HDAC9* is a protein coding gene that belongs to the histone deacetylase superfamily, class IIA. It is located on chromosome 7p21.1, 915.4 kb in size and encodes a protein responsible for histone deacetylation [12]. The polymorphisms of *HDAC9* gene affect the acetylation and deacetylation processes of histones and thus further cause the activation or inactivation of certain genes [12, 13]. The most common polymorphism of the *HDAC9* gene is rs2107595, located in the 3′ region of the *HDAC9* gene.

So far, HDAC9 has been reported to affect several aspects of the pathogenesis of atherosclerosis, i.e. cholesterol efflux, platelet aggregation, interleukin 6 (IL-6) signaling, macrophage function, inflammation progression and vascular calcification [6, 11, 12, 14]. Moreover, several studies have found an association between the rs2107595 polymorphism of the *HDAC9* gene and the onset and progression of carotid atherosclerosis [12], ischemic and hemorrhagic stroke [15, 16], large vessel atherosclerotic stroke (LVAS) [14], and atherosclerotic coronary artery disease [17]. However, the data on the rs2107595 polymorphism and its association with the progression of carotid atherosclerosis are still limited.

In this study, we aimed to investigate the association between the rs2107595 polymorphism of the *HDAC9* gene and advanced carotid artery disease in a Slovenian cohort. The second aim was to investigate the effect of the above-mentioned SNPs on the expression of the *HDAC9* gene within the endarterectomy specimens obtained by surgery.

## Materials and methods

### Patients

In a present case-control study we enrolled 619 unrelated Caucasians, 311 consecutive patients with advanced carotid atherosclerosis (internal carotid artery (ICA) stenosis > 75%) and history of stroke/transitory ischemic attack (cases), and 308 control subjects. The control group encompassed consecutive subjects examined at the outpatient cardiology department for routinely planned cardiovascular risk assessment, so we include subjects of both genders without symptomatic carotid artery disease, i.e. either without any kind of ultrasound detectable atherosclerotic changes or subjects with mild atherosclerotic changes, however, the grade (percentage) of stenosis of common carotid artery (CCA) or ICA had to be hemodynamically nonsignificant, i.e. less than 50%. The cases enrolled into the study were revascularized, either by performing carotid surgery or with carotid stent implantation. Cases and control subjects were recruited from 3 Slovenian Health Care facilities, Medical Centre Medicor d.d. Ljubljana, Izola General Hospital and University Clinical Center Maribor, in the period from 2010 to 2019.

The degree of stenosis was determined by duplex vascular ultrasound examination and, if necessary for clinical purposes / reasons, also computed tomography (CT) angiography of the carotid arteries was applied. Ultrasound examinations and CT angiographies were performed by six specialists (three cardiologists and three radiologists) from the aforementioned institutions. The vascular ultrasound examinations consist of quantitative measurements of intima media thickness (IMT), the presence, type, and thickness of atherosclerotic plaques, blood flow rate, and assessment of the narrowing rate of CCA, ICA, and external carotid artery (ECA). The IMT on the left and right sides of CCA, ICA, and ECA were presented as the arithmetic mean of the three measurements [18, 19].

General information and history of risk behaviours, as well as anthropometric, clinical and laboratory parameters measured, such as gender, age, systolic and diastolic blood pressure, smoking status, alcohol, glucose, physical activity level, body mass index (BMI), cholesterol, high density lipoprotein cholesterol (HDL-C), low density lipoprotein cholesterol (LDL-C), triglycerides, high-sensitivity C-reactive protein (CRP), waist circumference, glycated hemoglobin (HbA1c), coronary artery disease (CAD), myocardial infarction (MI), duration of arterial hypertension (AH), and duration of diabetes mellitus (T2DM), were taken from cautiously taken health medical records.

To avoid the potential influence of confounding factors, patients with carotid artery stenosis of non-atherosclerotic origin, patients with neck tumor, patients with aortic arc stenosis and right artery subclavian stenosis, as well as patients with incomplete data, were not enrolled in the study. The study was approved by the National Medical Ethics Committee and was designed in accordance with the Declaration of Helsinki. After informative consent was signed, a detailed interview and physical examination were performed.

### Genotyping

Deoxyribonucleic acid (DNA) was isolated from peripheral blood leukocytes in the laboratory of the Institute of Histology and Embryology, Faculty of Medicine, University of Ljubljana. Isolation of genomic DNA was performed on a QIAcube apparatus (Qiagen GmbH, Hilden, Germany) according to the “V3” protocol, using a commercially available QIAamp DNA Blood Mini Kit (250) (Qiagen GmbH, Hilden, Germany), which included five different reagents (AL buffer, 96% ethanol, AW1 buffer, AW2 buffer, AE buffer) and an appropriate amount of protease (Qiagen GmbH, Hilden, Germany) (285 μL / 200 μL blood). Following all the steps from the manufacturer’s instructions, 3–12 μg of genomic DNA, i.e. 30–40 ng / μL, was isolated from 200 μL of blood.

The rs2107595 polymorphism was genotyped using TaqMan SNP Genotyping assay (Applied Biosystems, Foster City, CA, USA), according to the manufacturer’s recommendations. Genotyping was carried out in both groups (cases and controls).

### Immunohistochemistry

Immunohistochemistry (IHE) staining was performed on 30 endarterectomy specimens obtained by surgery. Formalin-xedpara n-embedded (FFPE) tissue sections of ICA were used for hematoxylin-eosin (HE) staining. Consecutive sections of 5 μm tissue were cut and then mounted and dried on glass slides from each paraffin block. Tissues were deparaffinized and dehydrated in graded alcohol solutions. The detection of HDAC9-positive cells was performed with the Novo Link Max Polymer Detection System (Leica Biosystems Newcastle Ltd., United Kingdom) following the manufacturer’s instructions. The slides were incubated with anti-HDAC9 monoclonal antibodies (TermoFisher, USA), diluted 1:100, overnight at 4 °C. Placental tissue was used as a positive, and tonsils as a negative control. The cells were defined as HDAC9-positive/negative. The area with HDAC9-positive cells was manually marked and numerical areal density of HDAC9-positive cells was calculated (the number of positive cells per mm^2^) [20, 21].

### Statistical analysis

Statistical analysis was performed using the SPSS, ver. 26.0, software for MS Windows (IBM SPSS, New York, USA). Normal distribution of data was checked using the Shapiro-Wilk test. Normally distributed continuous clinical data are presented as a mean ± SD, while non-normally distributed data (continuous variables) are presented as a median and interquartile range. Categorical variables are presented as numbers and percentage of patients affected. Normally distributed continuous clinical data were compared with an unpaired Student-t test, non-normally distributed continuous data were compared with Mann – Whitney U test. Discrete variables were compared by chi-square test. All variables where significant differences were obtained by univariate analysis (*p* < 0.05) were analyzed by logistic regression analysis. The deviation from Hardy-Weinberg equilibrium (HWE) was assessed by chi-square goodness of fit test.

## Results

History data, general anthropometric and laboratory characteristics of cases (subjects with ICA stenosis > 75%) and controls (subjects with no carotid atherosclerosis or ICA stenosis < 50%) are presented in Table 1. There were no significant differences between the groups with respect to BMI, HDL-C levels, triglycerides, HbA1c, coronary artery calcium (CAC) score, presence and duration of T2DM, and duration of AH. On the other hand, statistically significant differences were found between the groups in the following parameters: age (*p* < 0.001), gender (*p* < 0.001), excessive alcohol consumption (*p* < 0.001), smoking (*p* < 0.001), waist circumference (*p* < 0.001), systolic blood pressure (SBP) (*p* < 0.001), diastolic blood pressure (DBP) (*p* < 0.04), total cholesterol (*p* < 0.001) and LDL-C levels (*p* < 0.001), hs-CRP (*p* < 0.001), AH (*p* < 0.001), CAD (*p* < 0.001) and MI (*p* < 0.033). Patients from the case group (with stenosis > 75%) were older, had higher waist circumference, and greater alcohol and tobacco smoking use than in comparison to the control group. Also, patients from the case group had higher SBP, DBP, higher total cholesterol and LDL-C levels, lower HDL-C levels, as well as significantly higher hs-CR*P* values compared to the control group.

**Table 1:** Anthropometric, clinical and laboratory characteristics of of the groups of cases and controls

Characteristics	Cases (311)	Controls (308)	*P* value
Number	311	308	
**Age (years)**	70.5 ± 8.5	62.9 ± 11.6	**< 0.001**
BMI (kg/m^2^)	28.3 ± 4.4	28.7 ± 4.3	0.42
**Waist (cm)**	102.2 ± 12.5	96.7 ± 14.7	**< 0.001**
**Male sex (%)**	208 (67.2)	148 (47.7)	**< 0.001**
**Systolic blood pressure (mm Hg)**	144.9 ± 20.0	136.6 ± 18.0	**< 0.001**
**Diastolic blood pressure (mm Hg)**	80.2 ± 10.6	83.0 ± 13.4	**0.04**
**Blood glucose level (mmol/L)**	5.9 (5.2–7.6)	5.3 (4.8–6.6)	**< 0.001**
**History of arterial hypertension (%)**	286 (91.5)	206 (66.9)	**< 0.001**
**Tobacco smoking (%)**	81 (26.0)	40 (13.0)	**< 0.001**
**Total cholesterol (mmol/L)**	4.5 ± 1.3	5.1 ± 1.2	**< 0.001**
**LDL cholesterol (mmol/L)**	2.4 (1.9–3.2)	2.9 (2.2–3.8)	**< 0.001**
HDL cholesterol (mmol/L)	1.2 ± 0.3	1.4 ± 0.4	0.14
Triglycerides (mmol/L)	1.4 (1.0–1.9)	1.4 (1.0–2.0)	0,61
HbA_1c_ (%)^a^	7.8 ± 1.3	7.2 ± 1.5	0,19
**hs-CRP (mg/L)**	3.0 (2.5–8.0)	1.5 (0.8–3.5)	**< 0.001**
Diabetes mellitus (%)	109 (34.5)	95 (30.9)	0.39
**Myocardial infarction (%)**	44 (13.8)	28 (8.6)	**0.03**

The values represent mean ± standard deviation. Values with parenthesis with the minus sign inbetween represent the median (1st quartile – 3rd quartile). Bold indicates statistically significant results

^a^The average value for haemoglobin A_1C_(HbA_1c_)

The distribution of genotype and allele frequency of the rs2107595 polymorphism of the *HDAC9* gene in the subjects from the case and control groups is shown in Table 2. By univariate analysis we found significant differences in genotype (*p* < 0.02) and allele (*p* < 0.01) frequencies between the case group and controls. Genotype distribution did not deviate significantly from Hardy-Weinberg equilibrium.

**Table 2:** Genotype and allele distribution of rs2107595 in cases and controls

	Cases (311)	Controls (308)	*p* value
AA (MAF)	20 (6.4%)	7 (2.3%)	
AG	105 (33.8%)	94 (30.5%)	**0.02**
GG	186 (59.8%)	207 (67.2%)	
A allele (%)	145 (23.3%)	108 (17.5%)	**0.01**
G allele (%)	477 (76.7%)	508 (82.5%)	
HWE	0.33	0.33	

*HWE* Hardy-Weinberg equilibrium

We used logistic regression analysis to assess whether rs21007595 polymorphism was independently associated with the progression of atherosclerosis after adjusting for age, waist circumference, tobacco smoking, SBP, DBP, total cholesterol, LDL-C levels, and hs-CRP. The results indicate the existence of a statistically significant association in two genetic models, codominant (OR 3.81; CI 1.06–13.77; *p = 0.04*), and recessive (OR 3.10; CI 1.16–8.27; *p = 0.02*) (Table 3).

**Table 3:** Logistic regression analysis adjusted for different confounders (age, waist, sex, SBP, DBP, history of hypertension, tobacco smoking, total cholesterol, LDL cholesterol, hs-CRP, MI, Blood Glucose level) according to the co-dominant, dominant and recessive genetic models

	***CASES/CTRLs***	***OR (95% CI)***	***p***†
**CODOMINANT (rs**2107595**)**			
AA vs. GG*(reference)	20/7 vs. 186/207	3.81 (1.06–13.77)	**0.04**
AG vs. GG*(reference)	105/94 vs. 186/207	1.21 (0.74–1.97)	0.45
**DOMINANT (rs**2107595**)**			
[AA + AG] vs. GG*(reference)	125/101 vs. 186/207	1.35 (0.85–2.16)	0.26
**RECESSIVE (rs**2107595**)**			
AA vs. [AG + GG]*reference	20/7 vs. 291/301	3.10 (1.16–8.27)	**0.02**

*OR* odds ratio, *CI* confidence interval

p† values - adjusted for age, waist, sex, SBP, DBP, history of AH, tobacco smoking, total cholesterol, LDL-C, hs-CRP, MI, blood glucose level

Within the endarterectomy sequesters obtained from surgically treated patients with advanced ICA, a statistically significantly higher numerical areal density of HDAC9-positive cells was found in subjects with the A allele in comparison with HDAC9 homozygotes (wild type) (755 ± 235/mm^2^ versus 392 ± 153/ mm^2^; *p* < 0.001).

## Discussion

In the present study we investigated the association between the rs2107595 of the *HDAC9* gene and advanced carotid atherosclerosis in a Slovenian cohort of subjects. Logistic regression analysis clearly showed a significant tendency of the carriers of the rs2107595 polymorphism towards an increased risk for the progression of carotid atherosclerosis in two genetic models (co-dominant and recessive). Anyway, the results obtained and showed above need further to be discussed, mainly from the aspect of their comparability with the to date published results of other similar research, but also from the point of view of their potential use in clinical practice, as well as the limitations of the present study.

Markus et al. [16] investigated the association of the rs2107595-*HDAC9* gene with LVAS, IMT, and asymptomatic carotid plaque in community samples. Also, they investigated the expression of HDAC9 in the cerebral and major arteries. They found an association of the rs2107595 polymorphism with IMT (*p* = 0.0018) as well as the presence of carotid plaque (*p* = 0.0022). Immunohistochemical analysis revealed the presence of HDAC9 in both endothelial and vascular smooth muscle cells. Also, Azghandi et al. [17] suggested that the risk allele of the *HDAC9* gene exerts its effects through the increased expression of HDAC9. They also highlighted the association of the *HDAC9* gene with the onset of atherosclerosis, heart attack and stroke [22]. Furthermore, Shroff et al. [14] investigated the influence of the rs2107595 *HDAC9* gene on the differences in leukocyte gene expression in patients with LVAS. LVAS patients with the risk allele and controls without it showed a statistically significant difference in the expression of HDAC9 (*p* < 0.05). Also, they suggested an association of rs2107595 of the HDAC9 with peripheral immune, lipid, and coagulation pathways in patients with LVAS [14]. These studies show that the role of the HDAC9 gene and its rs2107595 polymorphism is one of the major causes of atherosclerosis and associated diseases. Specifically, HDAC9 is at a key position in the pathophysiological pathways leading to the formation of atherosclerotic plaque. Our findings are in line with this study and also suggest an association of rs2107595 polymorphism with the progression of carotid atherosclerosis, as well as an increased risk of manifest cerebrovascular events in Slovenian subjects.

Opposite to our study, Su et al. [23] in the Chinese Han population found no association of the rs2107595 polymorphism and the IS onset in any of the genetic models (all *p* > 0.05) [23]. Comparing these results to the European population we have to consider the potential influence of different environmental and epigenetic factors. We did not find any other papers which would had published the results opposite to ours.

Most studies which found the high expression of HDAC9, highlight its role in the onset and progression of atherosclerosis, large vessel atherosclerotic stroke, increased risk of stroke and heart attack, etc. Therefore, there is also a growing body of research highlighting the importance of using selective and non-selective HDAC9 inhibitors in the treatment and prevention of atherosclerosis, heart attack, and stroke. With this regard, the study performed by Brookes et al. [22] emphasizes the importance of HDAC9 in the pathogenesis of stroke, as well as the possible positive effect of using sodium valproate (as a non-selective HDAC9 inhibitor) in reducing the recurrence rate of stroke (*p = 0.002*) [19]. HDAC9-selective inhibitors are still in the experimental testing stages, and the agent A4291 has the best forecasts so far [24].

However, we have to report also several important limitations related to our study. First of all, the number of subjects included was relatively small what influences the validity and reliability of the results obtained. Second, the study was conducted on an ethnically homogeneous population of a small society what significantly limits its generalizability. Furthermore, we processed only one HDAC9 gene polymorphism, so we cannot exclude the potential influence of other important polymorphisms. In general, it is widely known that there are many potential SNPs that might significantly influence the risk of the ASCVD development. Here we have to acknowledge the already discussed and published fact that currently only the multifactorial/multiSNPs approach (with the consequent potential of construction of reliable and validates polygenic risk scores) can be widely recommended [25–27].

Anyway, the results of our study suggest the potential of the use of rs2107595 polymorphisms determination in order of refined assessment of the risk for advanced atherosclerotic carotid disease in clinical settings, not only in patients with already established ASCVD in different vascular beds, e.g. in patients with coronary or peripheral arterial disease, but also in subjects presented with no manifest disease. Furthermore, we can assume the potential applicability also in close relatives of patients with advanced carotid disease, with the aim of potential adjustments of the level of applied preventive therapies. The confirmation of the existence of the rs2107595 polymorphism can guide the treatment towards much more aggressive approach, with more strict advice and monitoring with regard to the therapeutic lifestyle changes and even lower target values of classical ASCVD major risk factors set (e.g. to insist in adjustment of the diet to decrease the systemic proinflammatory state, as well as earlier to achieve preferably recommended blood pressure and the lowest possible levels of atherogenic blood lipids, primarily of LDL-C). However, we need more research on larger samples to assure the necessary validity of the predictive value of the existing rs2107595 polymorphism to be used as a true ASCVD risk modifier.

*In conclusion*, we demonstrated an association of the rs2107595 polymorphism of the HDAC9 gene with the advanced carotid artery disease in Slovenian cohort. We speculate that the mechanism of action of the HDAC9 rs2107595 polymorphism might be via the increased expression of HDAC9 in atherosclerotic plaques. Further research with a larger sample should elucidate the role of rs2107595 in the onset and progression of ASCVD as such and the potential serious clinical consequences that accompany it, such as ischemic stroke.

## Data Availability

The datasets used and/or analysed during the current study are available from the corresponding author on reasonable request. All the data presented (and according sources) within the paper are fully available for a review.

## Abbreviations

AH: Arterial hypertension
ASCVD: Atherosclerosis related cardiovascular disease
BMI: Body mass index
CAC: Coronary artery calcium
CAD: Coronary artery disease
CCA: Common carotid artery
CT: Computed tomography
DBP: Diastolic blood pressure
DNA: Deoxyribonucleic acid
ECA: External carotid artery
FFPE: Formalin-xedpara n-embedded
HbA1c: Glycated hemoglobin
HDAC9: Histone deacetylase 9
HDL-C: High density lipoprotein cholesterol
hs-CRP: high-sensitivity C-reactive protein
HWE: Hardy-Weinberg equilibrium
ICA: Internal carotid artery
IHE: Immunohistochemistry
IL-6: Interleukin 6
IMT: Intima media thickness
IS: Ischemic stroke
LDL-C: Low density lipoprotein cholesterol
LVAS: Large vessel atherosclerotic stroke
MI: Myocardial infarction
rs2107595: rs2107595 single nucleotide polymorphism
SBP: Systolic blood pressure
SNP: Single nucleotide polymorphism
T2DM: Diabetes mellitus type 2
TIA: Transitory ischemic attack
